# Danggui Shaoyao San ameliorates Alzheimer’s disease by regulating lipid metabolism and inhibiting neuronal ferroptosis through the AMPK/Sp1/ACSL4 signaling pathway

**DOI:** 10.3389/fphar.2025.1588375

**Published:** 2025-04-09

**Authors:** Kai Gong, Shuang Zhou, Li Xiao, Mengzhen Xu, Yuhe Zhou, Kaihui Lu, Xin Yu, Jiang Zhu, Chuanguo Liu, Qingjun Zhu

**Affiliations:** ^1^ Innovative Institute of Chinese Medicine and Pharmacy, Shandong University of Traditional Chinese Medicine, Jinan, China; ^2^ College of Traditional Chinese Medicine, Shandong University of Traditional Chinese Medicine, Jinan, China; ^3^ Affiliated Hospital of Shandong University of Traditional Chinese Medicine, Jinan, China; ^4^ Experimental Center, Shandong University of Traditional Chinese Medicine, Jinan, China; ^5^ Key Laboratory of Traditional Chinese Medicine Classical Theory, Ministry of Education, Jinan, China

**Keywords:** Alzheimer’s disease, neuron ferroptosis, AMPK/Sp1/ACSL4 pathway, Danggui Shaoyao San, lipid metabolism

## Abstract

**Introduction:**

Alzheimer’s disease (AD) is a neurodegenerative disorder characterized by cognitive decline; recent studies suggest that neuronal ferroptosis plays a key role in its pathogenesis. Danggui Shaoyao San (DSS), a traditional Chinese medicine formula, has shown demonstrated neuroprotective effects, but its precise mechanisms in AD treatment remain unclear. This study aims to investigate the mechanism of DSS in treating AD by inhibiting neuronal ferroptosis, explore whether DSS alleviates AD by suppressing neuronal ferroptosis via the AMPK/Sp1/ACSL4 pathway.

**Methods:**

Chemical composition of DSS was identified by LC-MS/MS, followed by network pharmacology to predict targets and pathways. Molecular docking assessed binding affinities between DSS compounds and key proteins (AMPK, Sp1, ACSL4). *In vivo* experiments on APP/PS1 mice evaluated DSS effects on cognitive function, oxidative stress markers, lipid peroxidation, and ferroptosis-related proteins.

**Results:**

Network pharmacology analysis suggested that DSS regulates lipid metabolism and inhibits neuronal ferroptosis via the AMPK pathway. Molecular docking revealed strong binding affinities between DSS compounds and AMPK downstream proteins, Sp1 and ACSL4. *In vivo* experiments showed that DSS improved cognitive function, enhanced antioxidant capacity, reduced lipid peroxide accumulation, and decreased Fe^2+^ content in brain tissue. Furthermore, DSS increased the expression of FTH, p-AMPK, and GPX4 while decreasing Sp1 and ACSL4 levels, thereby inhibiting ferroptosis.

**Conclusion:**

DSS alleviates AD symptoms by suppressing neuronal ferroptosis via the AMPK/Sp1/ACSL4 axis, representing a novel lipid metabolism-targeted therapeutic strategy.

## 1 Introduction

Alzheimer’s disease (AD) is a devastating neurological disorder that primarily affects the elderly (Sharma et al., 2020; Zhong et al., 2023). AD, a progressive neurodegenerative disorder affecting over 9.83 million elderly people in China alone (Lee et al., 2009), poses an escalating challenge to global healthcare systems due to limited therapeutic options. Current pharmacological interventions only alleviate symptoms with significant side effects, and emerging stem cell therapies remain limited by safety concerns, including tumorigenic risks (Vissers et al., 2019). This therapeutic impasse underscores the urgent need to explore novel pathogenic mechanisms, particularly those involving metabolic dysregulation. Recent advances implicate ferroptosis—an iron-dependent cell death pathway characterized by lipid peroxidation—as a pivotal contributor to AD pathogenesis (Dixon et al., 2012; Ashraf et al., 2020; Jiang et al., 2021). The unique susceptibility of the mammalian brain to ferroptosis stems from its high polyunsaturated fatty acid (PUFA) content coupled with AD-specific pathological features: iron accumulation in Aβ-plaque regions (Ayton et al., 2020; Ayton et al., 2021), elevated lipid peroxidation products near neurofibrillary tangles (Benseny-Cases et al., 2014), and reduced antioxidant defenses (GSH/GPX4) (Gleason and Bush, 2021; Sato et al., 2022).

Emerging evidence places AMP-activated protein kinase (AMPK), a master regulator of cellular energy homeostasis, at the intersection of AD pathology and ferroptosis regulation. AMPK dysfunction in AD correlates with impaired mitochondrial biogenesis and disrupted lipid metabolism, culminating in neuronal death (Assefa et al., 2020; Chew et al., 2020; Barone et al., 2021). Crucially, AMPK activation exerts anti-ferroptotic effects by suppressing PUFA peroxidation (Dixon et al., 2012; Fu et al., 2016), possibly through downstream modulation of acyl-CoA synthetase long-chain family member 4 (ACSL4). This rate-limiting enzyme catalyzes the incorporation of PUFA into membrane phospholipids, generating ferroptosis-prone phosphatidylethanolamine species (Doll et al., 2017; Long and Holtzman, 2019; Wang et al., 2022; Hacioglu et al., 2024). Interestingly, a regulatory axis susceptible to AMPK modulation is established by transcriptional activation of ACSL4 mediated by specific protein 1 (Sp1) (Li et al., 2019a). AMPK phosphorylation attenuates Sp1 nuclear translocation (Du et al., 2019; Zhang et al., 2019; Liu et al., 2020; Liu et al., 2024), thereby disrupting Sp1-ACSL4 promoter binding (Li et al., 2019a)and the subsequent lipid peroxidation cascade (Zhang et al., 2022; Zhou et al., 2023). Although this AMPK/Sp1/ACSL4 signalling has been implicated in models of cancer (Zhang et al., 2019; Liu et al., 2020; Liu et al., 2024), it remains unexplored in AD-associated ferroptosis.

A traditional Chinese medicine prescription called Danggui Shaoyao San (DSS) comes from Zhang Zhongjing’s Synopsis of the Golden Chamber. *Angelicae Sinensis Radix*, *Paeoniae Radix Alba*, *Atractylodes Macrocephala Rhizoma*, *Alismatis Rhizoma*, *Poria*, *Chuanxiong Rhizoma.* are among the six plant species that make up this mixture. Patients with mild to severe AD have showed significant improvements in their daily self-care abilities, mental health, and cognitive function when DSS is used clinically (Fu et al., 2016; You et al., 2020). While traditional Chinese medicine formulations such as DSS demonstrate neuroprotective efficacy in AD clinical trials (Fu et al., 2016; Huang et al., 2020; Yang et al., 2021), their mechanistic interplay with ferroptosis-related pathways remains elusive. Our study investigates the hypothesis that DSS ameliorates AD pathology via AMPK/Sp1/ACSL4 axis-mediated regulation of neuronal lipid metabolism and ferroptosis suppression, using the APP/PS1 transgenic mouse model.

## 2 Materials and methods

### 2.1 Analysis of network pharmacology

Potential targets of DSS were identified using the TCMSP and Swiss Target Prediction databases (www.swistargetprediction.ch). The GeneCards database (www.genecards.org) and the DisGeNET database (www.disgenet.org/home) provided AD-related targets. After retrieving ferroptosis-related targets from the FerrDb V2 database, 116 ferroptosis-related genes were selected as final targets for further investigation. To determine the PPI association, 83 overlapping targets associated with drug-treated diseases were loaded into the STRING database. This process yielded potential disease related targets. The common targets associated with disease and DSS were found using Faith Born (http://www.bioinformatics.com.cn), GO and the KEGG enrichment study. This study investigated the mechanism of action of DSS on the AMPK/Sp1/ACSL4 pathway. We found the structure of three proteins (PRKAA1, PRKAA2, Sp1) from the PDB database (https://www1.rcsb.org/), and the structure of ACSL4 protein from the AlphaFold Protein Structure Database (https://alphafold.ebi.ac.uk), and performed molecular docking with the predicted top ten compounds to check their docking energy. Based on binding energy references, we screened combinations of small molecules and proteins with low binding energy. Finally, PyMOL 2.5.0 software was used to visually present the docking results.

### 2.2 Preparation of DSS

All herbal materials were purchased from Anguo Runde Pharmaceutical Co., Ltd. and identified by Professor Qingjun Zhu of Shandong University of Traditional Chinese Medicine. *Angelica sinensis (Oliv.)* (Smoke-dried root of Danggui, Angelicae Sinensis Radix), *Paeonia lactiflora Pall* (Sun-dried root of Baishao, Paeoniae Radix Alba), *Ligusticum chuanxiong Hort* (Oven-dried rhizome of Chuanxiong, Chuanxiong rhizome), *Atractylodes macrocephala Koidz* (oven- or sun-dried rhizome from Baizhu, Atractylodis macrocephalae rhizome), *Poria cocos (Schw.) Wolf* (dried sclerotia of Fuling, Poria) and *Alisma plantago-aquatica Linn* (dried stem tuber of Zexie, Alismatis rhizoma) were mixed in a ratio of 3:16:4:4:8:8. The herbs were extracted for 1 h after soaking for half an hour in 1:8 (w/v) distilled water. After collecting the filtrate, distilled water was added for a second extraction at a ratio of 1:6 (w/v). To prepare a DSS extract with a concentration of 3 g/mL at 60°C, the two filtrates were mixed and concentrated. The drug extract was mixed evenly twice and then filtered through five layers of gauze to collect the filtrate; finally, the extraction liquid was concentrated to 1 g/mL using a rotary evaporator and stored in the refrigerator at −20°C.

### 2.3 LC-MS/MS analysis

Chromatography was conducted using an ACQUITY UPLC ^®^ HSS T3 (2.1 × 100 mm, 1.8 µm) (Waters, Milford) with a column maintained at 40°C. The flow rate and injection volume were set at 0.3 mL/min and 2 μL, respectively. For LC-ESI (+)-MS and LC-ESI (−)-MS analyses, the mobile phases were 0.1% formic acid in acetonitrile (B2) and 0.1% formic acid in water (A2) and acetonitrile (B3) and 5 mM ammonium formate in water (A3), respectively, and the gradient elution procedures were: 0–1 min, 8% B2/B3; 1–8 min, 8%–98% B2/B3; 8–10 min. 98% B2/B3; 10–10.1 min, 98%–8% B3; 10.1–12 min, 8% B2/B3.45. Using an ESI ion source, the Q Exactive (Thermo Fisher Scientific) mass spectrometer was used to detect metabolites. The positive and negative ion mode voltage was 3,500 V/−2,500 V, the dry gas temperature was 325°C, sheath gas pressure, 40 arb; aux gas flow, 10 arb. The metabolites were identified by accuracy mass and MS/MS data which were matched with HMDB, massbank, LipidMaps, mzcloud, KEGG and the metabolite database bulid by Panomix Biomedical Tech Co., Ltd.

### 2.4 Animals and treatments

Two strains of 6-month-old male mice, APP/PS1 and C57BL6/J, were used in this experiment. Both were obtained from Hangzhou Ziyuan Experiment Co., Ltd. (No. SCXK (Zhe)-2019-0004). The Animal Care Committee of SDUTCM gave its approval to all studies (authorization number: No. 20230303001). The treatments included Danggui Shaoyao San (DSS) at low dose (7.5 g/kg), middle dose (15 g/kg), and high dose (30 g/kg), as well as donepezil (DOP) at 3 mg/kg. Behavioral tests were conducted after 30 days of continuous dosing. After the behavioral tests, the mice were euthanised with carbon dioxide and their brain tissue was harvested for subsequent analysis.

### 2.5 Behavioral test

The Novel Object Recognition Test (NORT) and the Morris Water Maze (MWM) are the two parts of the behavioral test. A popular behavioral test called the NORT takes advantage of rodents’ innate curiosity about novel stimuli. The assessment of learning and memory functions in rodents with intact memory is made possible by the fact that they will investigate new objects longer than familiar ones (Lueptow, 2017). The set-up consisted of a square box made of white, non-porous plastic, several objects of different shapes and colors, and a photo-capturing device. The experiment consisted of three phases: habituation, familiarisation and testing. The first day was spent familiarising the mice with their new environment. On the second day, they were shown two similar objects and their exploration times were recorded. On the third day, one object was replaced with a brand new one. The mice were then placed in the box with their backs to the wall and any objects. The exploration times for the novel object B (TB) and the familiar object A (TA) were recorded for a period of 5 min. Calculation formula: RI $=\frac{TB}{\text{TA}+\text{TB}}$ .

The MWM is used to evaluate learning capacity and spatial memory (Nunez, 2008). The main body of the water maze is a circular barrel-shaped pool with a diameter of 1.2 m and a height of 40 cm, and a camera acquisition and analysis device was connected to the top of the pool. In the localisation and navigation test, all mice had to reach the hidden platform, and the MWM device automatically tracked the mice’s swim path and the time they reached the hidden platform (escape latency). After four training sessions per day for four consecutive days, the mice’s escape latency was recorded again. The researcher then took away the hidden platform and counted how many times the mice went back to the original platform location as well as how long they stayed there. These tests assessed the mice’s spatial recall and recollection of the platform’s placement.

### 2.6 Immunofluorescence (IF)

After behavioral testing, each group of mice was euthanised. Mouse brain tissue was removed after intracardiac perfusion with pre-cooled 0.9% saline. For 24 h, the mouse brain tissue was preserved in 4% paraformaldehyde (PFA) at 4°C. Brain tissue was sectioned at 30 μm thickness using a cryostat (Leica, CM 3050) in preparation for tissue immunofluorescence.

To prevent non-specific binding, 10% BSA in 1x PBS was blocked for 60 min at room temperature after the OCT was removed from the sections by three 1x PBS washes. After application of the primary antibody, the sections were kept at 4°C overnight. The next day, the sections were treated with the appropriate secondary antibodies (Alexa Fluor 488 or Alexa Fluor 594) for 2 h at room temperature after three washes in 1x PBS. Afterward, the sections underwent three PBS washes before being mounted using DAPI-containing anti-fade mounting solution. Nikon A1 confocal microscope was used to take pictures of each segment. A Nikon A1 confocal microscope was used to image each section.

### 2.7 Western blot (WB)

Mouse hippocampal tissue was homogenised and processed using ice-cold RIPA lysis buffer containing protease and phosphatase inhibitors. Protein concentrations in each sample were determined using a BCA kit (Vazyme, E112). Equal amounts of protein from each group were loaded onto 10% SDS-PAGE gels and proteins were transferred to a PVDF membrane at 200 mA for 60 min. The membrane was blocked with 3% BSA in TBST for 2 h, followed by overnight incubation at 4°C with primary antibodies against phosphorylated AMPK (Affinity, AF3423), AMPK (Affinity, AF6423), ACSL4 (Affinity, DF12141), SP1 (Affinity, AF6121), FTH (Affinity, DF12141), GPX4 (Abcam, Ab125066) and β-actin (Beyotime, AF003). After five washes with TBST, the membrane was incubated with a secondary antibody for 2 h at room temperature. The blot was visualised by ECL and the grey values were quantified using ImageJ 1.8.0 software.

### 2.8 Real-time PCR

The kit is used to extract tissue RNA (Vazyme, RC101). The RNA is reverse transcribed to complementary deoxyribonucleic acid (cDNA) (Vazyme, Q323) and amplified using the primers listed in Table 1 of the Bio-Rad CFX 96 System (Bio-Rad Laboratories, Hercules, CA, United States). The PCR includes the following steps: initial denaturation is carried out at 95°C for 30 s, followed by 40 cycles at 95°C for 10 s and 60°C for 30 s. The primers are listed in Table 1.

**TABLE 1:** Primer sequences for use in qRT-PCR.

Target	Forward primer	Reverse primer
FTH	AAC​CAG​CGA​GGT​GGA​CGA​A	CAA​TGA​AGT​CAC​ATA​AGT​GGG​GA
ACSL4	CCT​GAG​GGG​CTT​GAA​ATT​CAC	GTT​GGT​CTA​CTT​GGA​GGA​ACG
GPX4	TGT​GCA​TCC​CGC​GAT​GAT​T	CCC​TGT​ACT​TAT​CCA​GGC​AGA
AMPK	ACC​TGA​GAA​CGT​CCT​GCT​TGA​TG	AAT​GAC​TTC​TGG​TGC​GGC​ATA​ATT​G
Sp1	ACC​CAC​AAG​CCC​AGA​CAA​TCA​C	TGG​AGG​AGA​GTT​GAG​CAG​CAT​TC
β-actin	GGC​TGT​ATT​CCC​CTC​CAT​CG	CCA​GTT​GGT​AAC​AAT​GCC​ATG​T

### 2.9 Enzyme-linked immunosorbent assays (ELISA)

Hippocampal tissues were removed from the −80°C refrigerator, weighed and recorded. Hippocampal tissue homogenates were prepared by placing different groups of hippocampal tissue in pre-labelled grinding tubes with PBS (tissue mg: PBS μL = 1:10) and grinding beads and fixed in a tissue homogeniser. The tissue homogenate was transferred to a new labelled EP tube and centrifuged at 5,000 rpm for 10 min. The supernatant was carefully collected and stored at 4°C as a reserve. The appropriate ELISA kits (BC 1985, BC0625, BC5335, BC5325, BC1310, BC1195, BC0205; Solarbio, S0131S; Beyotime, E-EL-0128c, E-BC-K773-M; Elabscience) were used to detect TC, TG, LDL-C, HDL-C, T-AOC, GSH-Px, CAT, MDA, 4-NHE and Fe^2+^ in hippocampal tissue according to the kit instructions.

### 2.10 Statistical analysis

All statistical analyses were performed using SPSS software (version 23.0, IBM, United States). Data were expressed as mean ± standard deviation (SD). Prior to statistical testing, data were assessed for normality using the Shapiro-Wilk test and for homogeneity of variance using Levene’s test. An independent samples t-test was used for comparisons between the Control and Model groups. If the data met the assumptions of normality and homogeneity of variance, an independent samples t-test was performed. Otherwise, a Mann-Whitney U test was used. For comparisons between the Model group and the four treatment groups (Low-dose, Middle-dose, High-dose DSS and positive drug group), one-way analysis of variance (ANOVA) was performed, followed by Dunnett’s *post hoc* test to compare each treatment group with the Model group. If the data were not normally distributed, a Kruskal–Wallis test was used, followed by Dunn’s multiple comparison test for *post hoc* analysis. A p-value <0.05 was considered statistically significant.

## 3 Results

### 3.1 Analysis of network pharmacology

**FIGURE 1:**
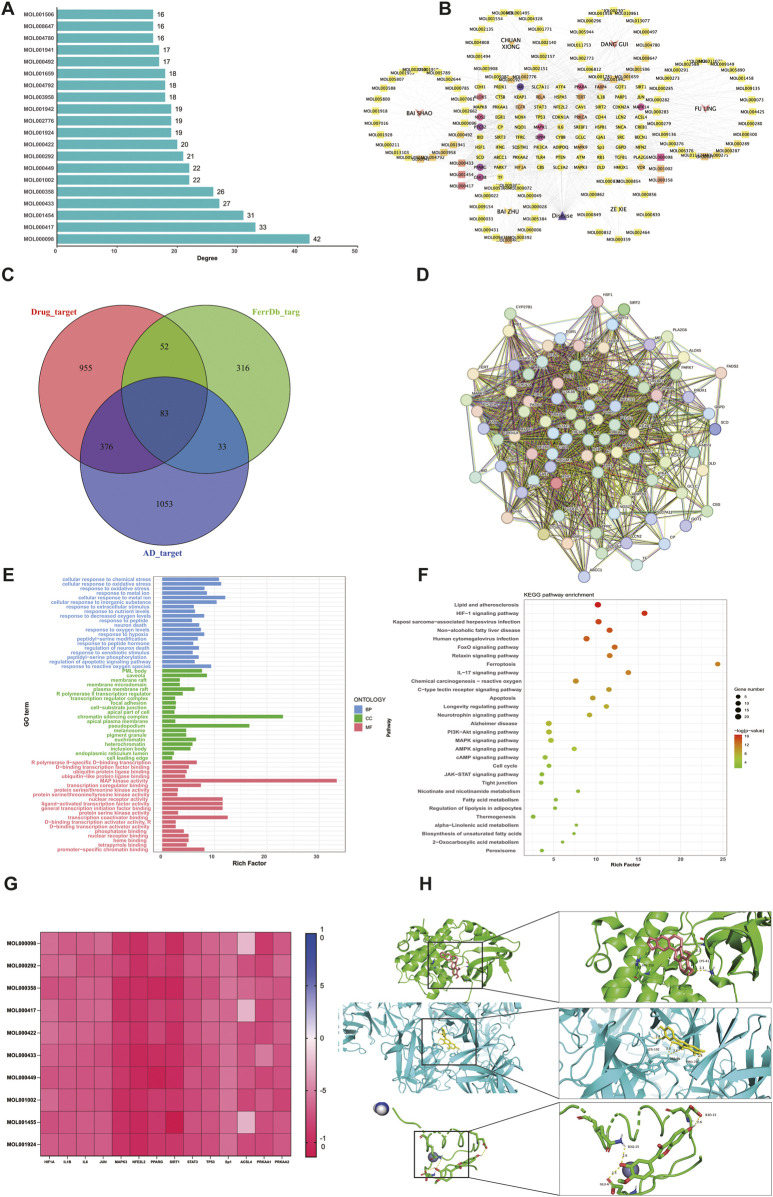
Analysis of network pharmacology **(A)** Summary of DSS main bioactive components **(B)** Drug-compound-target-disease network diagram **(C)** Active component and illness target Venn diagram **(D)** Protein-protein interaction network of common targets of DSS, AD and ferroptosis. **(E)** GO functional enrichment analysis **(F)** Analysis of KEGG pathway enrichment **(G)** Top active ingredient, Top target and AMPK pathway interface energy heat map **(H)** AMPK1‐Quercetin Specifies the connection result, AMPK2‐Calycosin Specifies the connection result and Sp1‐Calycosin Specifies the connection result.

A total of 112 active compounds and 1,466 targets were identified from DSS using the prediction results of the TCMSP and Swiss Target Prediction platforms. Using “Alzheimer’s Disease” as the keyword, disease-related targets were obtained from the GeneCards and DisGeNET databases. Gene names were standardized and deweighted. Additionally, Targets associated with ferroptosis were acquired from the FerrDb V2 database. AD and ferroptosis-related genes were combined as the final list of genes for the disease. The top 20 compounds in terms of connectedness (Figure 1A) were determined based on degree connectivity by generating a drug-compound-target-disease network plot (Figure 1B). The potential disease targets were then intersected, and a Venn diagram was created showing the overlap between targets from different databases was created (Figure 1C). To generate a protein-protein interaction (PPI) network, the 83 overlapping targets were uploaded to the STRING platform (Figure 1D). These genes were then analyzed using KEGG and GO, and it was found that the mechanism of action of the drug was most likely related to the PI3K-Akt, MAPK and AMPK pathways (Figures 1E, F). We have a strong interest in the energy-sensing signalling pathway in AD patients, so we chose the AMPK signalling pathway for subsequent molecular docking and experimental validation. We selected the top 10 compounds to dock with AMPK and the downstream proteins of AMPK, Sp1 and ACSL4 proteins, and the docking energies were low, suggesting that there may be multiple compounds that can make the AMPK/Sp1/ACSL4 pathway work (Figure 1G). Display partial docking results using PyMOL 2 (Figure 1H).

### 3.2 Constituent analysis of DSS

**FIGURE 2:**
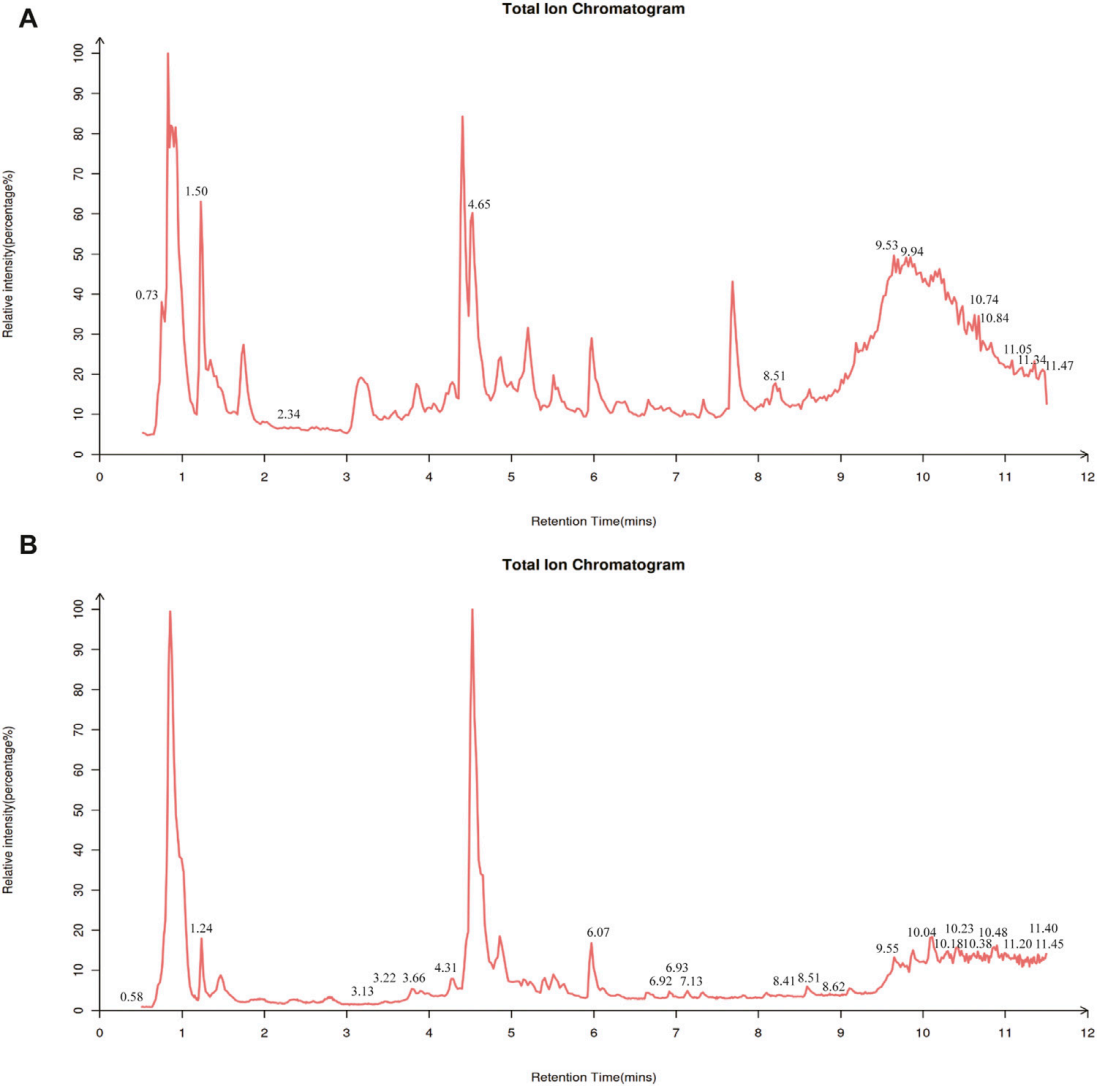
Constituent analysis of DSS. Total ion chromatogram of DSS in negative ion mode **(A)** and positive ion mode **(B)** of LC-MS/MS.

Considering that DSS alleviates AD, a thorough examination of its components might be helpful in determining the mechanism behind DSS’s therapeutic actions. Using LC-MS/MS analysis, the components of the DSS were identified. The findings showed that 236 compounds were present in the DSS, including fatty acids, carboxylic acids, organooxygen compounds, benzene and its substitutes, prenol lipids, steroids, and their derivatives. The thorough results are displayed in Supplementary Table S1 and Figure 2.

### 3.3 DSS can enhance APP/PS1 mice’s cognitive and learning memory performance

**FIGURE 3:**
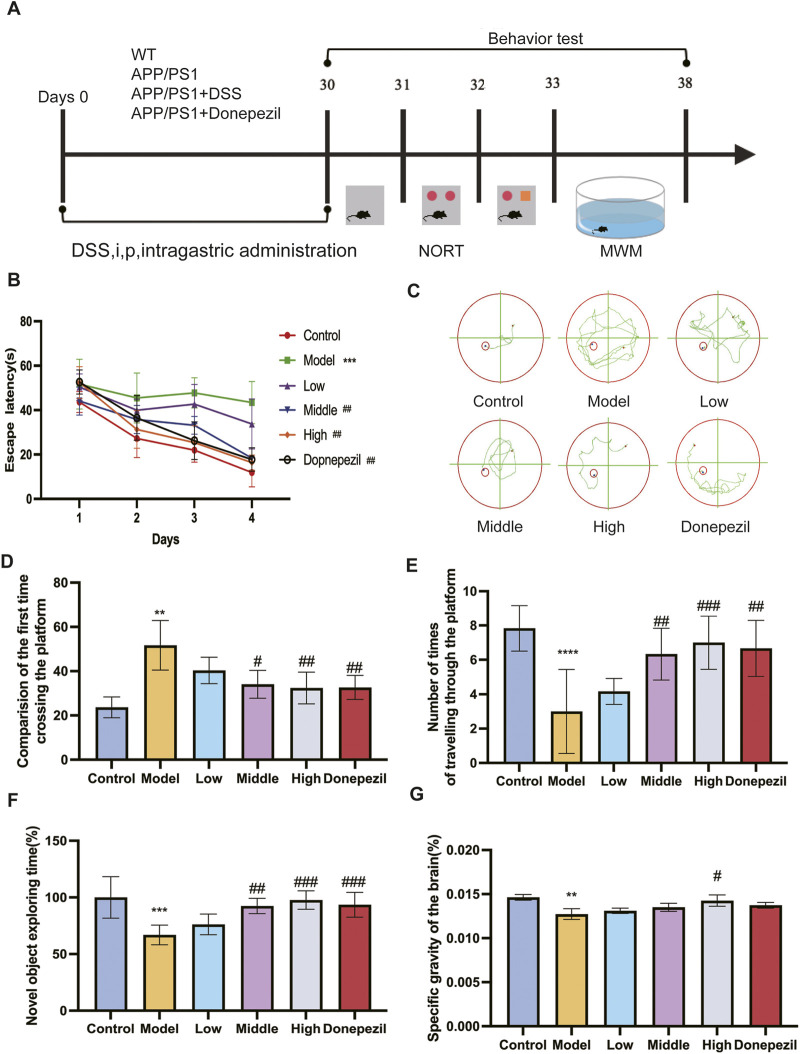
DSS can enhance APP/PS1 mice’s cognitive and learning memory performance. **(A)** The experiment’s implementation schedule. **(B)** The MWM test’s latency for the mice swimming to the platform. **(C)** On the fourth day, the track plot of all groups of mice in MWM test with platform. **(D)** Group comparison of the time taken by each group of mice to cross the platform. **(E)** Times in the MWM test with platform target quadrant where the platform was situated. **(F)** Comparison of NOR index in each group of mice. **(G)** Brain weight ratio of mice in each group. **p < 0.01, ***p < 0.001, ****p < 0.0001 vs. Control group, #*p* < 0.05, ##*p* < 0.01, ###*p* < 0.001 vs. Model group. (n = 6/ group).

We investigated the effects of DSS administration at different concentrations on memory and cognitive function in APP/PS1 transgenic mice. As shown in Figure 3B, there was significant change in escape latency in the Model group compared to the Control group over 4 days of MWM testing, suggesting learning and memory impairment in the Model group. The Middle and High DSS treatment groups showed significantly reduced escape latencies compared to the Model group. Figure 3C shows the swim trajectories of the mice in each group on day 4 of the Orientation Sailing experiment. It can be clearly seen that the swimming paths of the mice in the Model group are much more complex than those of the other groups. The first time across the platform was significantly delayed in the Model group compared to the Control group (Figure 3D). The Low DSS group showed a marked improvement, while the Middle and High DSS groups showed improved performance compared to the Model group. The Model group crossed the platform fewer times than the Control group as shown in Figure 3E. The Middle and High DSS groups showed significantly more platform crossings than the Model group, whereas the Low DSS group did not show significant differences compared to the Model group. The exploration time for novel objects was significantly lower in the Model group compared to the Control group (Figure 3F), reflecting deficits in object recognition memory. The Low, Middle and High DSS groups showed significant improvements in exploration time compared to the Model group, with the High DSS group showing the most robust improvement. Compared to the Control group, the brain percentage of mice in the Model group decreased significantly and the statistical difference was significant (Figure 3G). The High-dose DSS group showed a statistically significant effect, and the brain proportions of the treatment and DSS-positive drug groups were higher than those of the Model group. This demonstrates that DSS can ameliorate the morphological changes caused by neuronal pathogenic damage in mouse brain tissue atrophy.

### 3.4 DSS enhances the antioxidant capacity and attenuates the accumulation of lipid peroxides in APP/PS1 mice, and has a protective effect on the brain

**FIGURE 4:**
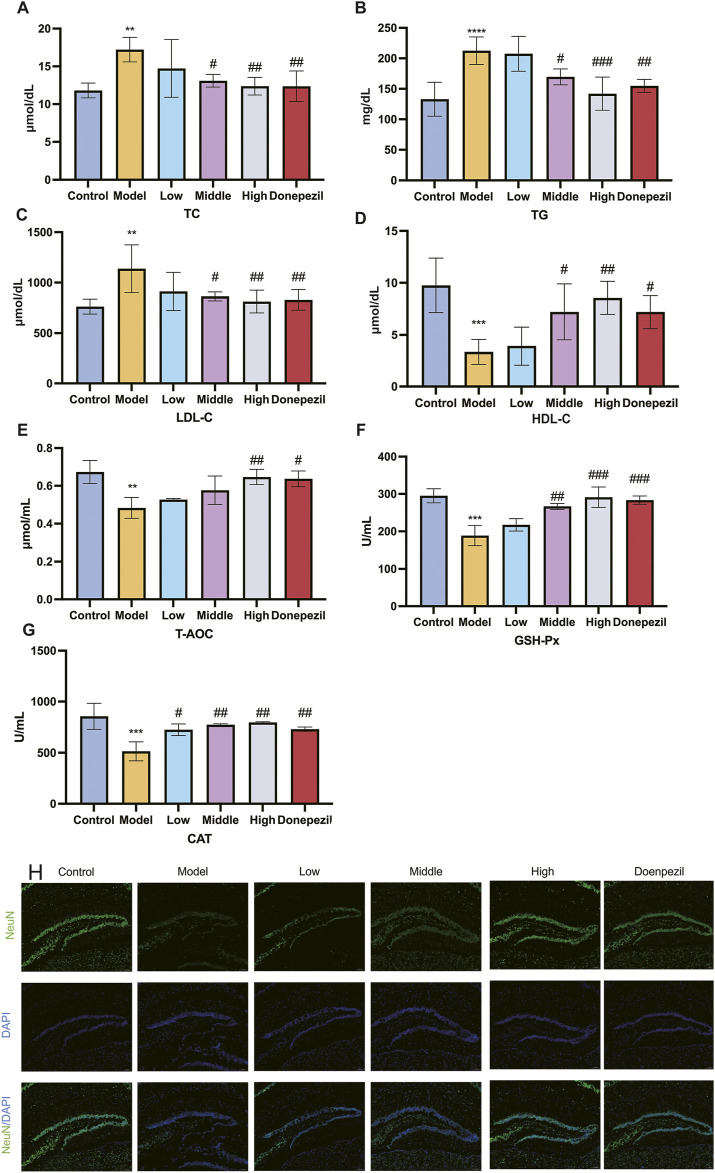
DSS enhances the antioxidant capacity and attenuates the accumulation of lipid peroxides in APP/PS1 mice, and has a protective effect on the brain. **(A–D)** After the action of the drug, the changes in the blood lipid level of each group of mice. **(E–G)** Alterations in the mice’s capacity to produce antioxidants in each group following the drug’s impact. **(H)** Representative images of NeuN immunofluorescence in mouse hippocampus. ^**^ *p* < 0.01, ^***^ *p* < 0.001, ^****^ *p* < 0.0001 vs. Control group, ^#^ *p* < 0.05, ^##^ *p* < 0.01, ^###^ *p* < 0.001 vs. Model group (n = 5/group).

Lipid metabolism and antioxidant capacity play a key role in ferroptosis, so we investigated lipid metabolism levels and antioxidant capacity in mouse brain tissue. TC, TG, LDL-C levels and HDL-C are important indicators of lipid metabolism levels. The Low, Middle and High dose and positive drug groups can significantly reduce the levels of TC, TG and LDL-C and increase the level of HDL-C after treatment with DSS and DOP respectively. There are clear statistical differences and the greatest effect is seen with the High dose of DSS (Figures 4A–D). It was demonstrated that DSS can Control lipid levels and slow the progression of AD disease in APP/PS1 mice. GSH-Px, T-AOC and CAT are important indicators of the body’s antioxidant capacity. The results of this study show that, compared with the Control group, the antioxidant capacity of the Model group is significantly reduced, the levels of GSH-Px, T-AOC and CAT are significantly reduced and the statistical difference is significant. Compared with the Model group, the antioxidant capacity of each drug administration group was increased to different degrees, and the levels of GSH-Px, T-AOC and CAT were significantly increased (Figures 4E–G). The effect of the High dose group of DSS is the most significant. It can be seen that DSS can effectively scavenge ROS in the serum of APP/PS1 mice, improve the antioxidant capacity and lipid metabolism of tissues, and reduce the occurrence of ferroptosis in brain tissues, which plays a role in preventing and controlling the development of AD.

NeuN is a neuron-specific RNA splicing protein, which is involved in neuronal development and differentiation and synaptogenesis, and NeuN damage is strongly associated with the development of AD. Immunofluorescence results showed that the number of NeuN-positive cells in APP/PS1 mice was significantly lower compared with the Control group, and the DSS (Low, Middle, and High) dose groups significantly increased the number of NeuN-positive cells compared with the Model group (Figure 4H). This shows that DSS can reduce the histological damage to hippocampal neurons in APP/PS1 mice, reduce neuronal loss and improve their learning and cognitive memory functions.

### 3.5 DSS can reduce ferroptosis of neurons in APP/PS1 mice

**FIGURE 5:**
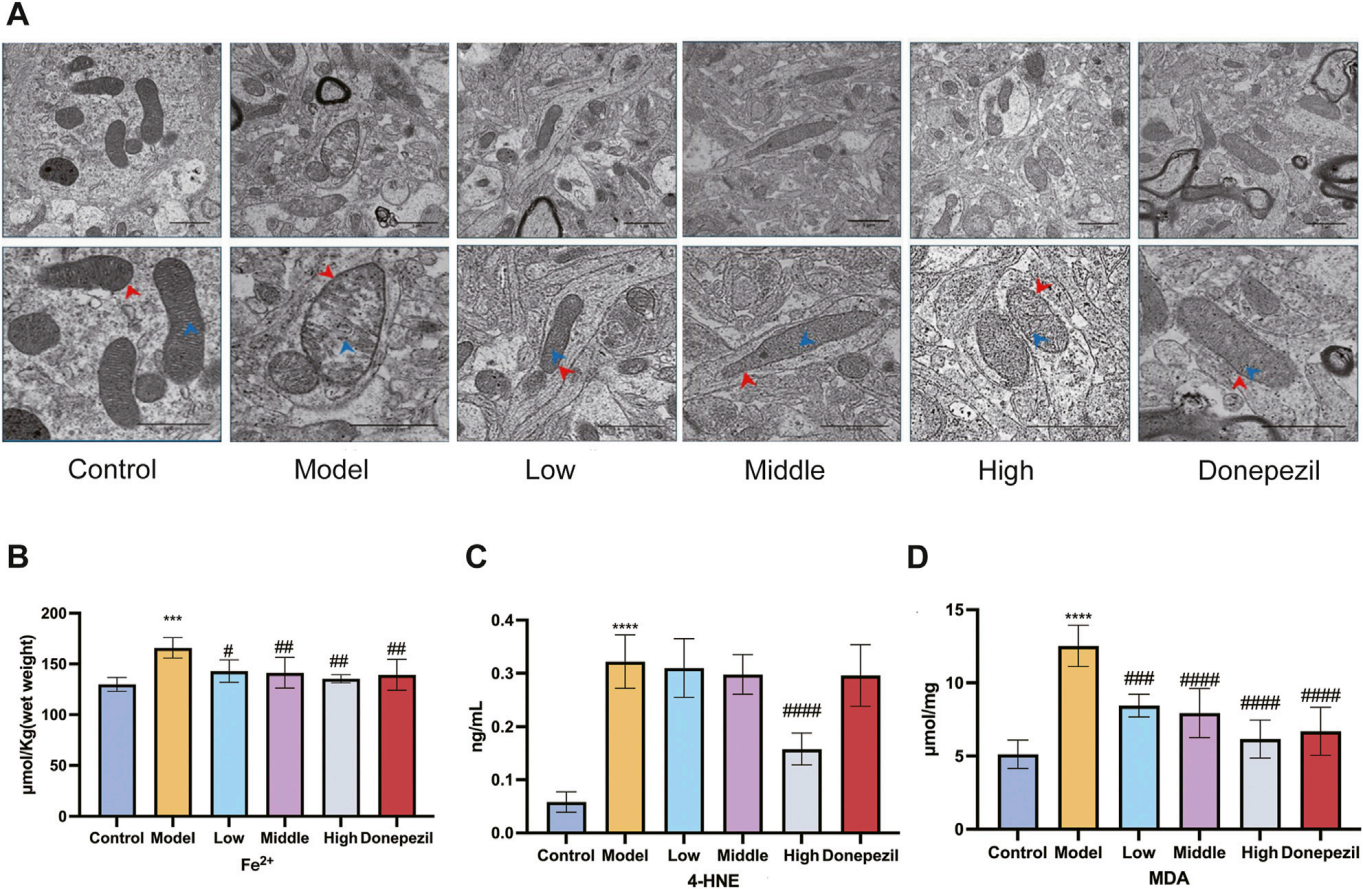
DSS can reduce ferroptosis of neurons in APP/PS1 mice. **(A)** Changes in iron content of hippocampal neurons in each group of mice. Scar bar: 1 μm in the upper middle picture of each group; The lower figure is 500 nm. **(B)** Changes in iron content of hippocampal neurons in each group of mice. Changes in 4-HNE **(C)** and MDA **(D)** contents of lipid peroxides in hippocampal neurons of mice in each group. ^***^ *p* < 0.001, ^****^ *p* < 0.0001 vs. Control group, ^#^ *p* < 0.05, ^##^ *p* < 0.01, ^###^ *p* < 0.001, ^####^ *p* < 0.0001 vs. Model group (n = 5/group).

The intracellular accumulation of iron ions during neuronal ferroptosis promotes the accumulation of lipid peroxides and the production of products such as MDA and 4-HNE. The concentration of iron ions and the relative amounts of MDA and 4-HNE may indirectly reflect the extent to which cellular ferroptosis occurs. Mitochondrial ultrastructure is the main morphological feature of ferroptosis in AD neurons. Hippocampal mitochondria from Control mice have a normal and regular shape, most are oval, their number is moderate, the structure of the ridge is clear and orderly, there are few breaks (blue scissors), the membrane is relatively complete (red arrows) and the boundary between structures is clear; the mitochondria of the hippocampal neurons of the mice in the Model group showed classic signs of ferroptosis, such as volume reduction and disappearance of the mitochondrial membrane, crest fracture, etc. After administration of DSS and DOP, mitochondrial damage was effectively alleviated in each drug dose group, and mitochondrial crest and membrane fracture were significantly reduced (Figure 5A). As we get older, iron continues to build up in the brain. This abnormal and active iron can easily lead to the degeneration of brain neurons and damage brain function. This is mainly due to the presence of highly oxidising Fe^2+^ in cells, which leads to the accumulation of large amounts of ROS and toxic lipid peroxides via the Fenton reaction, resulting in cell death. DSS can reduce the iron content of neurons in the hippocampal region of mouse models, with the effect being most significant at High doses (Figure 5B). Meanwhile, the expression of MDA and 4-HNE, markers of cellular ferroptosis, was also reduced by DSS, especially at High doses (Figures 5C, D). The results showed that DSS had positive effects on iron content and lipid peroxides in neurons.

### 3.6 DSS reduce neuron ferroptosis in APP/PS1 mice through the AMPK/Sp1/ACSL4 pathway

**FIGURE 6:**
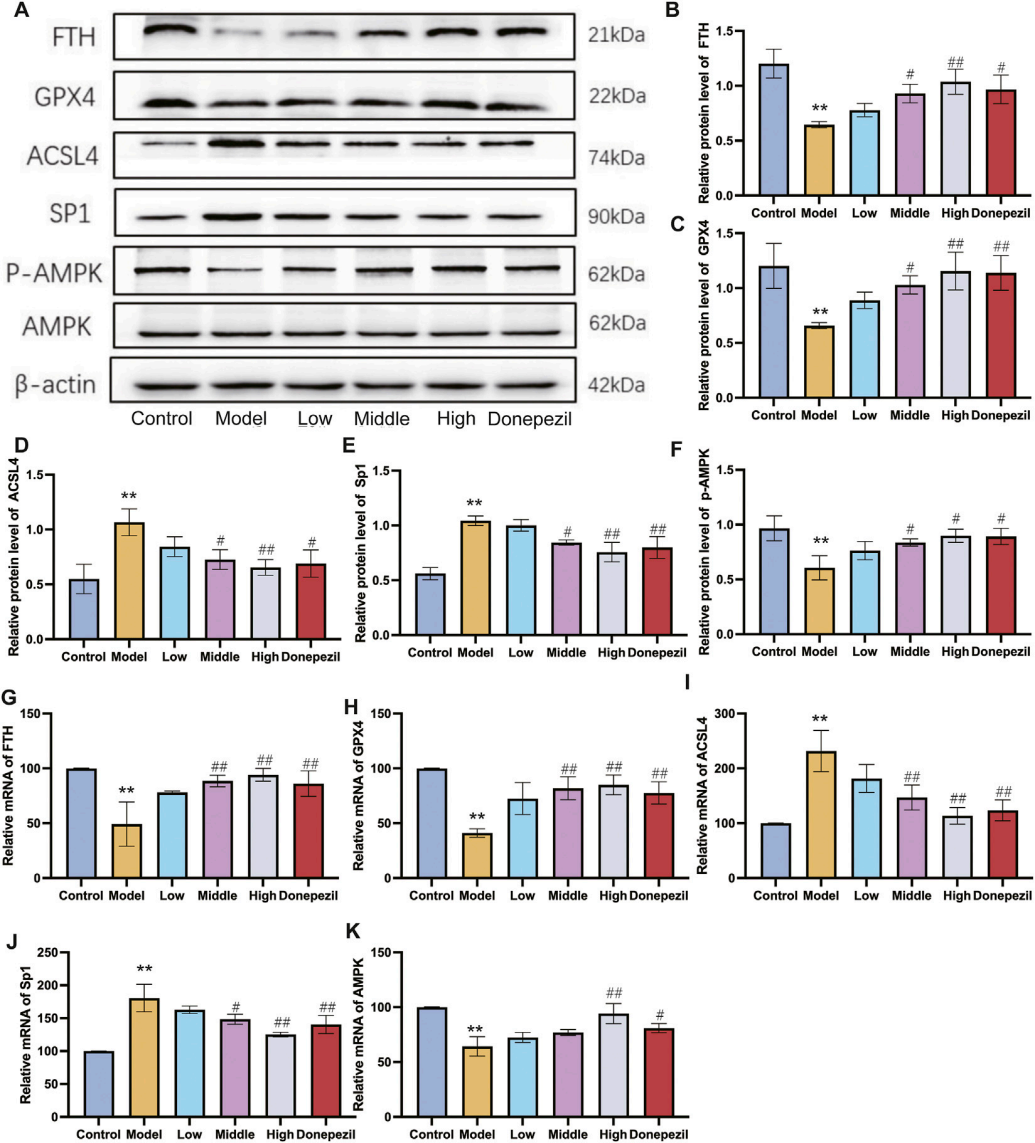
DSS reduce neuron ferroptosis in APP/PS1 mice through the AMPK/Sp1/ACSL4 pathway. **(A)** Representative Western blot results for FTH, GPX4, ACSL4, Sp1 and p-AMPK expression. **(B–F)** Graphs showing Western blot results of all mice examined **(G–K)** RT-qPCR analysis for FTH, GPX4, ACSL4, Sp1 and p-AMPK. β-actin was used as a loading control. ^**^ *p* < 0.01 vs. Control group, ^#^ *p* < 0.05, ^##^ *p* < 0.01 vs. Model group (n = 3/group).

**FIGURE 7:**
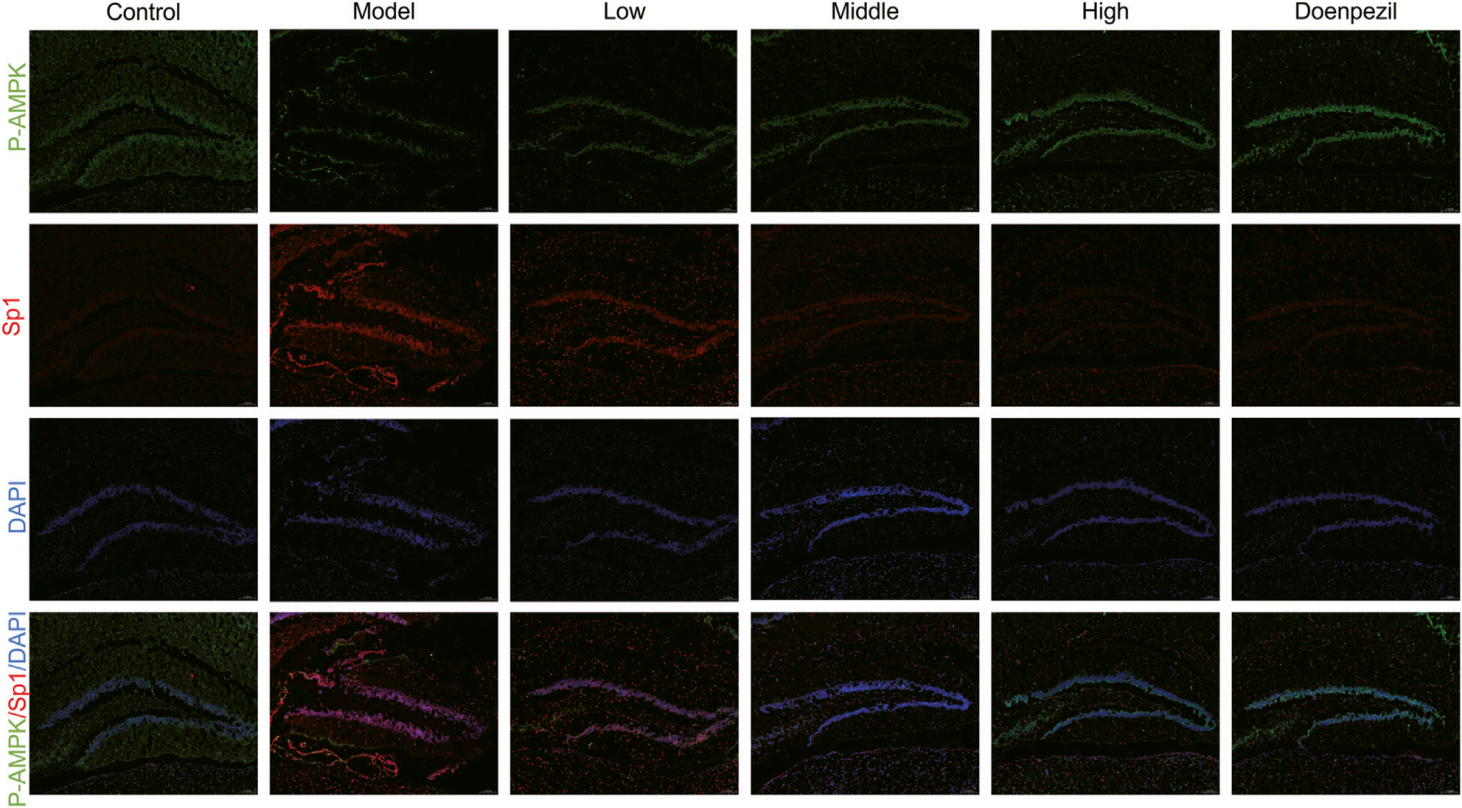
Representative immunofluorescence results of hippocampus tissue. Sp1 (red) and p-AMPK (green) immunofluorescence staining in the hippocampal regions with DAPI (blue) (n = 3/group).

Ferroptosis is controlled by the AMPK signalling pathway. Activation of AMPK has the ability to prevent ferroptosis from occurring. The downstream gene of the AMPK pathway is the transcription factor Sp1. It is highly expressed in AD and Sp1 can enhance ACSL4 transcription by binding to the ACSL4 promoter region. By regulating various unsaturated fatty acid conversions during lipid metabolism, ACSL4 has the ability to induce ferroptosis in neurons, causing damage to the nervous system. Ferroptosis is also important in cell metabolism and disease progression and is thought to be significantly regulated by GPX4 and FTH. In this study, the hippocampal expression of Sp1 and ACSL4 was increased in the APP/PS1 mice, whereas the expression of FTH, p-AMPK and GPX4 was downregulated compared to the Control mice. The expression of FTH, p-AMPK and GPX4 in hippocampal tissue from mice in each dose group increased with dose following DSS and DOP treatment, and the markers of ferroptosis, Sp1 and ACSL4, decreased with dose, reducing neuronal ferroptosis (Figures 6A–F). Similar results were observed by Immunofluorescence and RT-qPCR analysis (Figures 6G–K). The immunofluorescence results were consistent, with APP/PS1 mice showing significant expression of Sp1 protein and a marked reduction in P-AMPK protein. After treatment with different doses of DSS, p-AMPK expression gradually increased while Sp1 expression gradually decreased. These results suggest that DSS treatment significantly enhances AMPK activation and that activated p-AMPK may play a role in reducing Sp1 protein expression (Figure 7).

It illustrates how DSS can selectively activate the expression of p-AMPK, thereby inhibiting the interaction between Sp1 and ACSL4 promoters, downregulating ACSL4 expression by mitigating oxidative damage caused by iron accumulation and lipid peroxidation in neurons, and ultimately enhancing FTH and GPX4 levels in the hippocampal regions of mice.

## 4 Discussion

**FIGURE 8:**
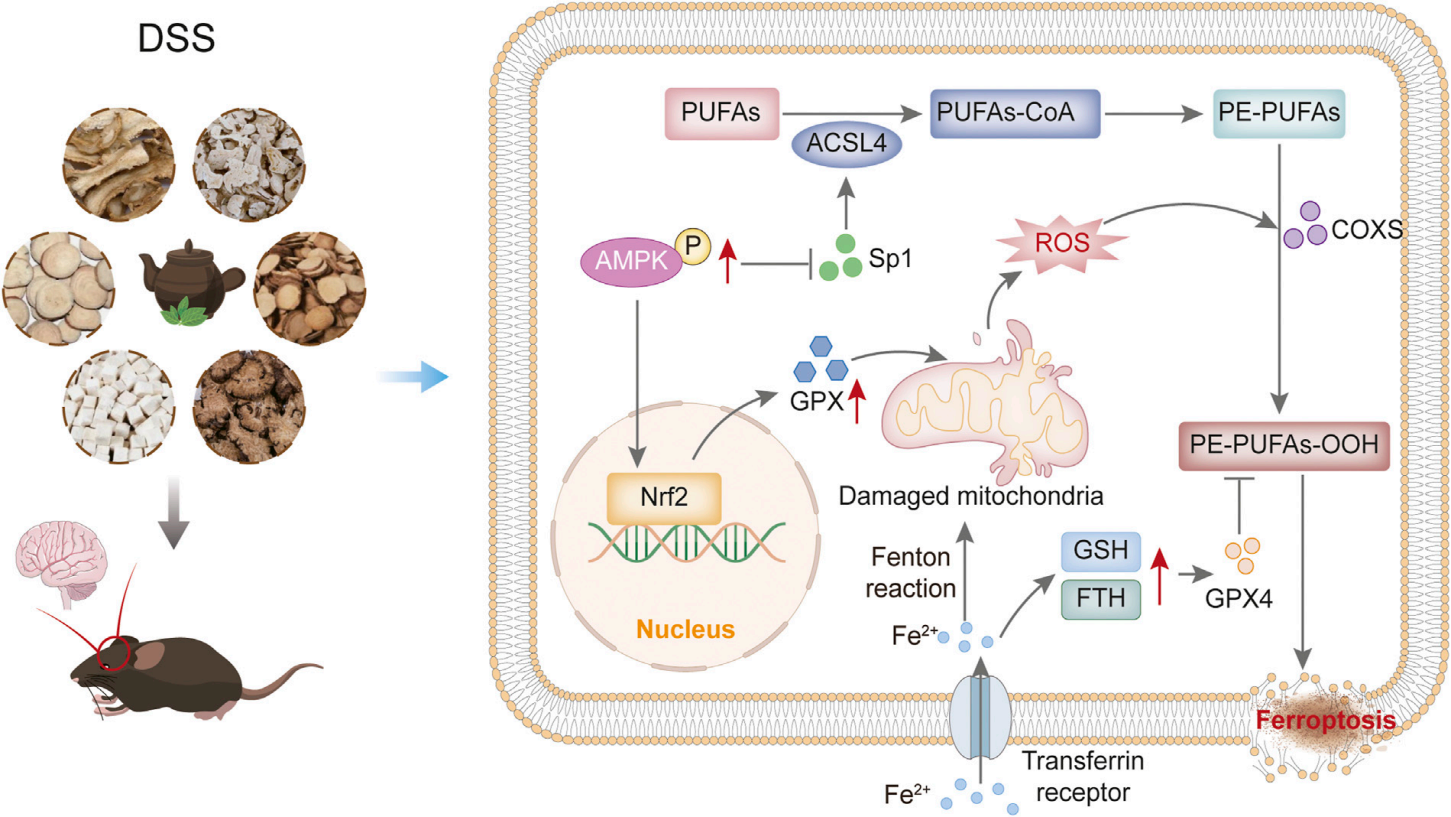
Mechanism of the impact of DSS on reduce neuron ferroptosis through the AMPK/Sp1/ACSL4 pathway in AD mice.

The present study demonstrates that DSS alleviates cognitive deficits in APP/PS1 mice by modulating the AMPK/Sp1/ACSL4 signalling axis to inhibit neuronal ferroptosis, providing a novel therapeutic strategy for AD. Behavioral assessments showed that DSS significantly improved spatial learning and memory in AD Model mice. Histopathological analysis also showed that both DSS and DOP attenuated neuronal atrophy and pathological damage. Mechanistically, DSS activated AMPK phosphorylation, suppressed Sp1-mediated ACSL4 transcription, reduced lipid peroxidation accumulation and upregulated ferroptosis inhibitors (FTH and GPX4), collectively restoring synaptic protein dynamics and neuronal homeostasis (Figure 8).

AD is characterized by Aβ deposition, neuroinflammation, and lipid metabolism dysregulation (Calsolaro and Edison, 2016; Selkoe and Hardy, 2016; Yamazaki et al., 2019). Based on network pharmacology approaches, this study identified 112 potential anti-AD active compounds, many of which have been experimentally validated for their therapeutic effects. Quercetin enhances neuronal antioxidant capacity by activating the PI3K/Akt and AMPK signaling pathways, effectively reducing pathological Aβ deposition (Khan et al., 2019; Cui et al., 2022). Berberine modulates ferroptosis-related pathways via the Nrf2/SLC7A11-GSH-GPX4 axis while simultaneously promoting Aβ clearance and suppressing neuroinflammation (Li et al., 2023; Sun et al., 2024). Ellagic acid exhibits multi-target neuroprotective effects, including inhibition of Aβ oligomerization, regulation of Tau protein hyperphosphorylation, enhancement of synaptic plasticity, and maintenance of mitochondrial homeostasis (Javaid et al., 2021; Zhu et al., 2022; Jha et al., 2024), Calycosin alleviates oxidative stress and inflammation in the hippocampus of AD model mice by activating the protein kinase C pathway, thereby improving cognitive function (Song et al., 2017). Additionally, steroidal compounds such as β-sitosterol and stigmasterol inhibit excessive microglial activation via the AMPK/NF-κB and AMPK/NLRP3 signaling pathways, thereby blocking the neuroinflammatory cascade (Ayaz et al., 2017; Song et al., 2017; An et al., 2022; Jie et al., 2022; Yesudas et al., 2023). Kaempferol, catechin, and moupinamide exert neuroprotective effects by modulating cholinergic system function and reducing oxidative stress (Thangnipon et al., 2012; Ide et al., 2018; Nejabati and Roshangar, 2022; Dong et al., 2023). Paeoniflorin inhibits neuronal ferroptosis by targeting the P53 protein, significantly reducing ROS levels (Zhai et al., 2023; Zhang et al., 2023). Baicalin improves cognitive function by regulating immunoglobulin-related protein expression and FADS1 succinylation modification, while Isoimperatorin exerts neuroprotective effects by simultaneously inhibiting neuroinflammation and oxidative stress (Zhao et al., 2024), Furthermore, folic acid supplementation has been reported to have beneficial effects on AD symptoms (Chen et al., 2021). Mechanistic studies suggest that DSS may intervene in AD pathology through a multi-component, multi-pathway, and multi-target regulatory network. At the molecular level, DSS modulates Aβ metabolism and Tau protein homeostasis; at the cellular level, it inhibits neuroinflammation and ferroptosis; and at the systemic level, it enhances synaptic plasticity and mitochondrial function. This multi-layered and multidimensional mechanism suggests that DSS holds potential as a multi-target therapeutic strategy for AD.

Ferroptosis, an iron-dependent form of regulated cell death driven by lipid peroxidation, has emerged as a critical contributor to neurodegeneration in early-stage AD (Gao and Jiang, 2018; Li et al., 2019b; Muhoberac and Vidal, 2019; Zhou et al., 2020; Raha et al., 2022). In AD brains, iron overload disrupts redox homeostasis via Fenton reactions, generating hydroxyl radicals that oxidise PUFAs in neuronal membranes. This process produces toxic lipid peroxides that damage cellular structures and trigger a vicious cycle of iron dysregulation and oxidative stress (Williams et al., 2006; Li et al., 2009; Ayala et al., 2014; Wang et al., 2017; Hu et al., 2021). Notably, our findings are consistent with recent studies highlighting ferroptosis as a therapeutic target, as iron chelators and antioxidants may alleviate neuronal death by interfering with this cascade (Li et al., 2019b; Zhou et al., 2020). In addition, as a member of the antioxidant enzyme family, GPX4 can effectively inhibit lipid peroxidation and convert phospholipid peroxides to non-toxic alcohols, thereby maintaining intracellular lipid balance and preventing ferroptosis (Scheerer et al., 2007; Borchert et al., 2018; Dar et al., 2024).

Dysregulation of lipid metabolism is a hallmark of AD, with PUFA-rich neuronal membranes being particularly susceptible to peroxidation. DSS exerts a dual protective effect by enhancing cellular antioxidant capacity through FTH-mediated iron storage and GPX4-dependent detoxification of lipid peroxides (Li et al., 2009; Wang et al., 2017; Hu et al., 2021; Yang et al., 2023; Dar et al., 2024), and suppressing ACSL4 transcription via AMPK/Sp1 signalling. AMPK, a master regulator of lipid metabolism, inhibits PUFA synthesis and ferroptosis (Dixon et al., 2012; Fu et al., 2016). We found that Sp1, a downstream effector overexpressed in AD, binds the ACSL4 promoter to drive the conversion of PUFAs into peroxidation-prone lipids (Li et al., 2019a; Long and Holtzman, 2019; Zhang et al., 2019; Liu et al., 2020). DSS disrupted this interaction by phosphorylating AMPK, thereby reducing Sp1 nuclear translocation and ACSL4 expression. Molecular docking analyses further suggested that DSS components have high affinity for AMPK and Sp1, providing a structural basis for their multi-target activity.

Unlike conventional single-target AD therapies, the multi-component nature of DSS simultaneously addresses iron dyshomeostasis, lipid peroxidation and synaptic dysfunction. This polypharmacological approach may overcome drug resistance and reduce the side effects associated with synthetic compounds. Importantly, DSS’s natural origin and low toxicity profile support its potential for long-term use in elderly patients. Our network pharmacology data further identified additional DSS components and pathways relevant to Alzheimer’s disease and ferroptosis, which warrant future validation.

While this study establishes DSS as a promising anti-ferroptotic agent, several limitations need to be noted. First, the exact bioactive compounds in DSS responsible for AMPK activation need to be isolated and validated. Second, it remains unclear whether DSS directly chelates iron or indirectly regulates iron transporters (e.g., transferrin receptor). Third, the long-term *in vivo* safety and blood-brain barrier permeability of DSS components need to be assessed. Future work should explore synergies between DSS and existing AD therapies, such as anti-amyloid agents, in combinatorial regimens.

## Data Availability

The original contributions presented in the study are included in the article/Supplementary Material, further inquiries can be directed to the corresponding authors.
