# Supplementary figures and images for: Danggui Shaoyao San ameliorates Alzheimer’s disease by regulating lipid metabolism and inhibiting neuronal ferroptosis through the AMPK/Sp1/ACSL4 signaling pathway
Source: Front Pharmacol. 2025 Apr 9;16:1588375. doi: 10.3389/fphar.2025.1588375 (PMC12014676; doi:10.3389/fphar.2025.1588375)

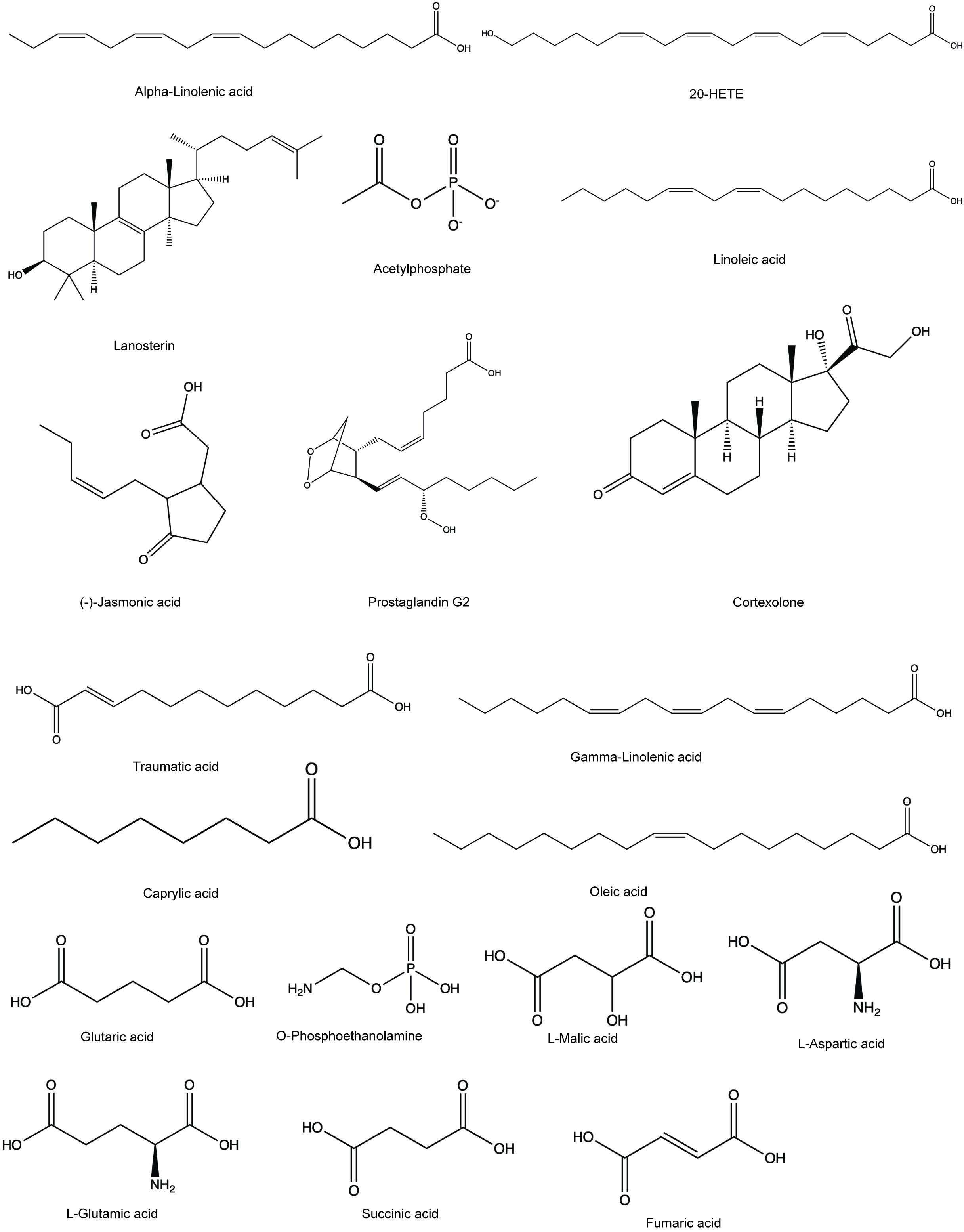

Supplement: Supplementary file 3 [file Image3.tif]

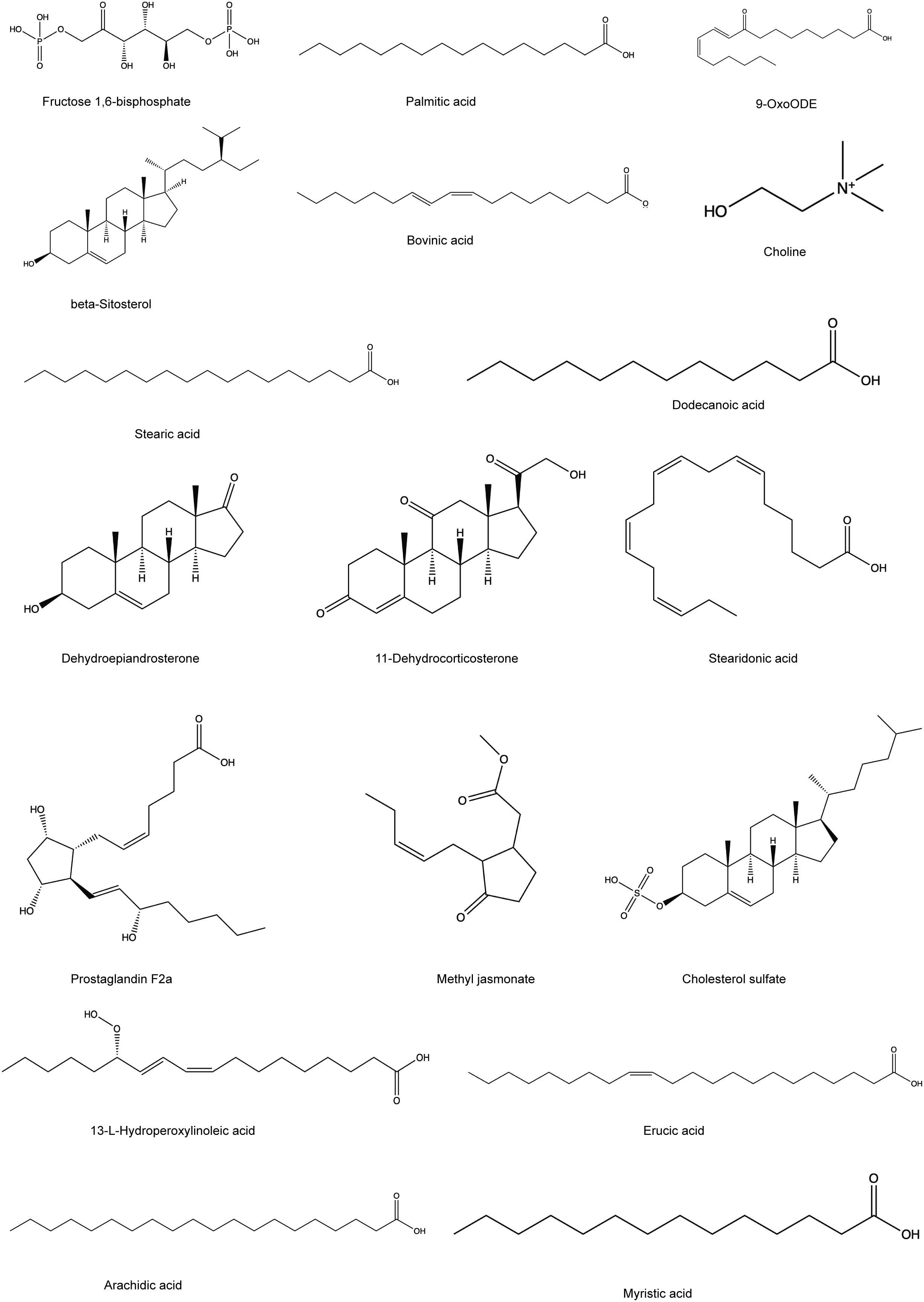

Supplement: Supplementary file 4 [file Image2.tif]

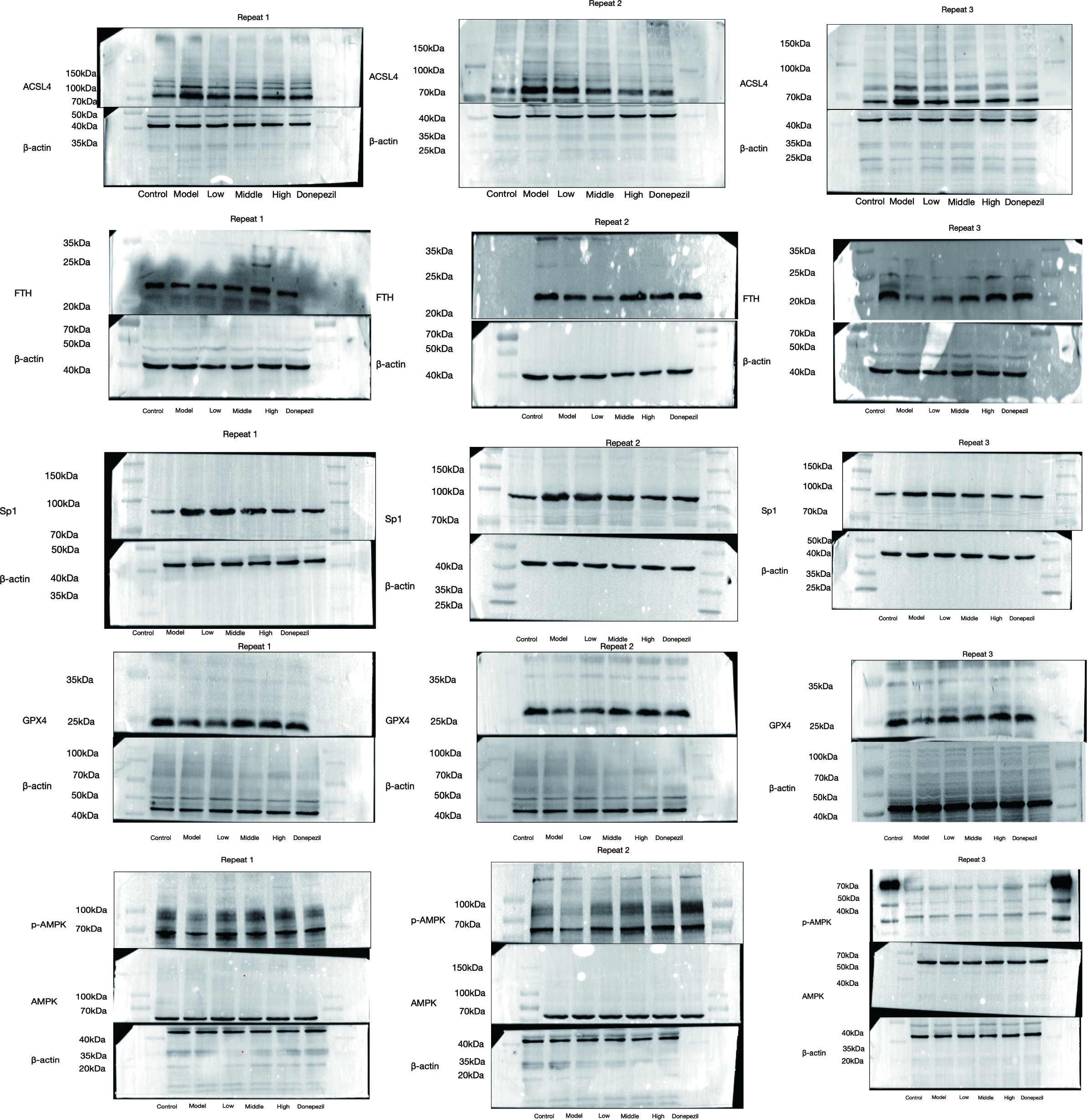

Supplement: Supplementary file 5 [file Image1.tif]
